# Cisplatin and Doxorubicin Induce Distinct Mechanisms of Ovarian Follicle Loss; Imatinib Provides Selective Protection Only against Cisplatin

**DOI:** 10.1371/journal.pone.0070117

**Published:** 2013-07-29

**Authors:** Stephanie Morgan, Federica Lopes, Charlie Gourley, Richard A. Anderson, Norah Spears

**Affiliations:** 1 Centre for Integrative Physiology, University of Edinburgh, Edinburgh, Scotland, United Kingdom; 2 Edinburgh Cancer Research Centre, University of Edinburgh, Edinburgh, Scotland, United Kingdom; 3 MRC Centre for Reproductive Health, University of Edinburgh, Edinburgh, Scotland, United Kingdom; Imperial College London, United Kingdom

## Abstract

**Purpose:**

Chemotherapy treatment in premenopausal women has been linked to ovarian follicle loss and premature ovarian failure; the exact mechanism by which this occurs is uncertain. Here, two commonly used chemotherapeutic agents (cisplatin and doxorubicin) were added to a mouse ovary culture system, to compare the sequence of events that leads to germ cell loss. The ability of imatinib mesylate to protect the ovary against cisplatin or doxorubicin-induced ovarian damage was also examined.

**Experimental design:**

Newborn mouse ovaries were cultured for a total of six days, exposed to a chemotherapeutic agent on the second day: this allowed for the examination of the earliest stages of follicle development. Cleaved PARP and TUNEL were used to assess apoptosis following drug treatment. Imatinib was added to cultures with cisplatin and doxorubicin to determine any protective effect.

**Results:**

Histological analysis of ovaries treated with cisplatin showed oocyte-specific damage; in comparison doxorubicin preferentially caused damage to the granulosa cells. Cleaved PARP expression significantly increased for cisplatin (16 fold, p<0.001) and doxorubicin (3 fold, p<0.01). TUNEL staining gave little evidence of primordial follicle damage with either drug. Imatinib had a significant protective effect against cisplatin-induced follicle damage (p<0.01) but not against doxorubicin treatment.

**Conclusion:**

Cisplatin and doxorubicin both induced ovarian damage, but in a markedly different pattern, with imatinib protecting the ovary against damage by cisplatin but not doxorubicin. Any treatment designed to block the effects of chemotherapeutic agents on the ovary may need to be specific to the drug(s) the patient is exposed to.

## Introduction

Premature ovarian failure (POF, also termed primary ovarian insufficiency; POI) is a common long-term adverse effect of chemotherapy treatment in premenopausal women [1] with consequences for both fertility and non-reproductive health such as osteoporosis [2] and cardiovascular disease [3]. The risk of developing POF is dependent on chemotherapy regimen [4], drug dosage [5], [6] and patient age [7]. Whilst it is well recognised that chemotherapy treatment can lead to POF due to loss of ovarian follicles, the exact mechanism by which this occurs is less certain [8]. Such knowledge is invaluable in the search to develop potential treatments to protect the ovary from chemotherapy-induced damage. The aim here is to determine the precise ovarian effects of two of the drugs commonly used to treat cancers in premenopausal women, cisplatin and doxorubicin.

By birth, the ovary has a fixed population of germ cells (oocytes) contained within follicles. These are formed prenatally at the primordial stage, consisting of an immature oocyte in meiotic arrest, surrounded by a few flattened somatic (granulosa) cells. Primordial follicles constitute the resting pool of female germ cells present for the duration of a female’s reproductive lifespan. At any one time, a small cohort is activated to grow, with the transition to the growing primary follicle stage marked by somatic cells becoming cuboidal and proliferating to fully surround the growing oocyte. Somatic cells continue to proliferate and form increasing numbers of layers around the oocyte, thecal cells are recruited from the interstitial stroma to surround the follicle and, as the follicle increases in size, fluid-filled patches form within the granulosa cell layers to create an antral cavity.

Numerous cell types in the ovary may be potential targets for damage by chemotherapeutic agents. It is often assumed that the primary cell type damaged is the oocyte within immature follicles, since ultimately loss of these leads to POF. There is, however, limited available evidence for this, as most studies showing oocyte damage used mature ovulated oocytes [9], [10], whereas in women, what is of importance is damage to oocytes contained within ovarian follicles. Within follicles, somatic cells could be the primary target [11], [12], leading to germ cell death and hence follicle loss indirectly. Also of importance is the follicle stage most at risk from chemotherapy-induced damage. Most studies examining follicles have focused on loss of primordial follicles [13], [14], as it is this that ultimately leads to POF. However, reduction of the primordial follicle pool could be due to either direct primordial follicle damage or to an indirect effect; damage to more mature, growing follicles would lead to increased recruitment of primordial follicles out of the resting pool and hence to premature depletion of that resting follicle reserve [8], [15]. Proliferating somatic cells within growing follicles also represent a more logical target for chemotherapy-induced damage than mitotically inactive cells. A better understanding of the mechanism by which chemotherapy-induced follicle loss occurs is vital for the development of potential protective treatments for those women at risk.

The work described here examines the effects of two chemotherapeutic drugs commonly used in the treatment of premenopausal women, cisplatin and doxorubicin. Cisplatin is a DNA cross-linking agent commonly used in the treatment of sarcomas and germ cell tumours. Its precise mechanism of action is not entirely clear, although it is known to intercalate with DNA strands causing crosslinking and adduct formation [16]. It is considered moderately gonadotoxic, and in a postnatal *in vitro* mouse ovary model can cause massive oocyte death [17]. Doxorubicin is an anthracycline which intercalates with DNA and prevents its replication and transcription [9]. It is used to treat a variety of cancers including breast cancer, lymphomas and leukaemias and recent evidence suggests that it is moderately ovotoxic [18]. Mature ovulated oocytes treated with doxorubicin undergo rapid DNA damage and cytoplasmic changes associated with apoptosis [10]; its action on immature follicles within the ovary is less clear although a recent study of human primordial follicles showed apoptotic damage to both oocytes and granulosa cells [19].

This work also investigates the ability of imatinib mesylate (called imatinib hereafter) to protect the ovary against damage induced by either cisplatin or doxorubicin. Imatinib is a tyrosine kinase inhibitor, used as the primary treatment for chronic myeloid leukaemia due to its inhibition of the tyrosine kinase BCR-ABL [20]. Imatinib can also inhibit c-Abl, PDGF receptor and c-kit [21], all of which can affect basic cellular function (cell signalling, proliferation and differentiation) including within ovarian follicles [22], [23]. Some recent work indicates that it has a protective effect against cisplatin-induced follicle loss [17], [24] although this has been disputed [25]. The ability of imatinib to protect against doxorubicin-induced ovarian damage is currently unknown.

The aim of this study was to investigate the mechanisms by which cisplatin and doxorubicin cause follicle loss using an *in vitro* system to culture mouse ovaries. The advantage of using such a culture controlled environment is that it allows precise determination of the cell type first damaged by the drugs while allowing follicles to develop in a highly physiological manner [26]. The system used here, culturing ovaries from newborn mice, supports follicle formation, growth initiation and development to the primary-secondary phase [27].

## Materials and Methods

### Animals

#### Ethics statement

This work was approved by the University of Edinburgh’s Local Ethical Review Committee. Animals were provided with food and water *ad libitum*, and kept in accordance with UK Home Office regulations. C57Bl6J mice were housed in an environmentally-controlled room on a 14-hour light, 10-hour dark photoperiod.

### Ovary Culture

Newborn female mice were culled by decapitation and ovaries dissected out into Leibovitz L-15 dissection medium (Invitrogen, Paisley UK) supplemented with 3 mg ml^−1^ bovine serum albumin (Sigma Aldrich Ltd, Dorset UK). Ovaries were cultured on Whatman Nucleopore membranes (Camlab Ltd, Cambridge UK; Whatman Nucleopore Polycarbonate Membranes 13 mm 8.0 µm) floating on 1 ml α-MEM medium (Invitrogen, Paisley UK) supplemented with 3 mg ml^−1^ bovine serum albumin (Sigma Aldrich), in a 24 well plate (Greiner Bio-one, Stonehouse UK) incubated at 37°C, 5% CO_2_. Information on follicle composition in control ovaries over the course of the culture period is shown in Figure S1.

After 24 h in culture (Day 1), medium was supplemented with varying doses of either cisplatin or doxorubicin-HCl, both from Sigma Aldrich, both first dissolved in sterile water (Day 2). Concentration ranges for each drug was determined from preliminary experiments finding the lowest dose leading to death of the majority of follicles; cisplatin was added to produce final concentrations of 0, 0.1, 0.5, 1 or 5 µg ml^−1^ and doxorubicin to produce final concentrations of 0, 0.01, 0.05, 0.1 or 0.2 µg ml^−1^. Following the 24 h of drug exposure (Day 2), ovaries were either snap-frozen for protein extraction, fixed for TUNEL analysis or moved into drug free culture for a further four days (Days 3–6), with 50% of medium changed every other day.

For the cultures containing imatinib mesylate (VWR International Ltd UK, dissolved in sterile water), imatinib was added to produce a final concentration of 3 µg ml^−1^. Dosage was determined from unpublished preliminary results indicating that this was the highest dose at which no significant morphological damage was seen when compared to control cultures. Imatinib was added to the medium on Days 1 to 3 of culture, with cisplatin or doxorubicin also added only on Day 2 of culture for 24 hours as in the previous experiments. Ovaries were then moved into drug-free culture for a further 3 days (Days 4–6) with 50% of medium changed every other day.

### Protein Extraction and Western Blotting

Frozen ovaries were homogenised in 20 µl lysis buffer (containing 50 mM HEPES buffer, 10% Triton X, 50 mM NaCl, Protease inhibitor cocktail and protease inhibitors [I and II], H_2_O; all purchased from Sigma Aldrich Ltd except for Protease inhibitor cocktail which was purchased from Roche Diagnostic Ltd) and centrifuged at 13000 rpm for 20 mins: supernatant was used for Western blotting. Approximately 10 µg of protein from each ovary was loaded onto a 7% acrylamide gel and run at 30 mA at room temperature. Protein was transferred onto a nitrocellulose membrane and blocked using 5% powdered milk (w/v) in phosphate buffered saline (PBS, pH7.3, 160 mM NaCl, 3 mM KCl, 8 mM Na_2_HPO_4_, 1 mM KH_2_PO_4_). PARP rabbit polyclonal antibody (New England Biolabs, Hertfordshire UK) was added at 1∶1000 dilution: this antibody to PARP detects both the full length (116 kD) and cleaved (89 kD) form. β-actin rabbit polyclonal antibody (Abcam, Cambridge UK) was added at 1∶5000 dilution as a loading control. Membranes were then incubated overnight at 4°C. After washing in PBS, membranes were incubated with Alexafluor anti-rabbit 750 (Invitrogen, UK) at 1∶2000 for 1 h, membranes re-washed and dried, and imaging and analysis carried out using a Li-cor scanner and Odyssey v1.2 software (Li-cor Biosciences, US). 89 kD bands were analysed to determine expression of Cleaved PARP, with 45 kD bands analysed to determine expression of β-actin.

### Histological Follicle Assessment

Ovaries cultured for six days were placed in Bouins fixative for 90 mins, paraffin wax-embedded, sectioned at 5 µm and stained with haemotoxylin and eosin. Every sixth section was photomicrographed and follicle assessment and counts carried out. All sections were assessed blind as to treatment. For the initial experiments with cisplatin and doxorubicin, 5 ovaries were assessed in each treatment group from 6 independent cultures. For the imatinib experiments, 7 ovaries were assessed for each treatment from 4 independent cultures.

A follicle was counted if the oocyte had a germinal vesicle present in the section analysed. All counted follicles were assessed for stage and health. For stage analysis, follicles were considered to be at the primordial stages where the oocyte was clearly associated with only pre-granulosa cells; the transitional stage where the oocyte was surrounded by granulosa cells some of which were flattened and some cuboidal; and at the primary stage where the oocyte was surrounded by a complete layer of cuboidal granulosa cells. Follicles were also classified by their morphological health, using standard criteria [28], [29]. In detail, a follicle was healthy if: a) the oocyte was round and contained evenly stained cytoplasm; b) there were no pyknotic granulosa cells (or no more than 1 for primary follicles); and c) there was clear attachment between the oocyte and its surrounding granulosa cells. Any follicle not considered healthy (not passing all three criteria) was further categorized as having an unhealthy oocyte only; unhealthy granulosa cells only; or having both oocyte and granulosa cells unhealthy. An oocyte was considered to be of poor health if it exhibited any one of shrunken cytoplasm, heavy or uneven eosin staining or no attachment between oocyte and its surrounding somatic cells. The assessment of the health of granulosa cells was dependent on follicle stage: for primordial and transitional follicles, a follicle was assessed as unhealthy if it contained any clearly pyknotic granulosa cell (out of the 3–6 present); for primary follicles, a follicle was assessed as unhealthy if it contained 2/3 or more clearly pyknotic granulosa cells (out of the 10–20 present). Abercrombie correction was applied to raw counts multiplied by frequency of section counted, in order to estimate total follicle number [30].

### TUNEL Analysis

Newborn mouse ovaries were placed in culture in control medium, or treated with medium supplemented with 1 µg ml^−1^ cisplatin or 0.1 µg ml^−1^ doxorubicin during Day 2 of culture. The second highest doses of chemotherapy drugs were used here to induce an appreciable level of damage, but not the overwhelming follicle loss seen at the highest doses. At the end of Day 2, ovaries were washed in PBS (Invitrogen, UK, pH7.4, 1.06 mM KH_2_PO_4_, 155.17 mM NaCl, 2.97 mM Na_2_HPO_4_.7H_2_O), fixed in 10% buffered formalin (Sigma Aldrich Ltd, UK) for 1 h, paraffin wax-embedded, sectioned at 5 µm and every 12^th^ section taken for analysis. After dewaxing, sections were permeabilised with 10 ug ml^−1^ proteinase K in 10 mM Tris/HCl, then labelled with TUNEL reagents according to manufacturer instructions (Roche Diagnostics Ltd). Sections were then counterstained with DAPI (Invitrogen, UK) and mounting medium applied (Vector Laboratories, Inc.). Sections were observed and images collected with a Leica AS6000 fluorescent microscope (Leica Microsystems, Germany). Sections were then washed in PBS and coverslips gently removed for subsequent haematoxylin and eosin staining. Sections were then re-observed and photomicrographs collected using a Leica DMLB microscope equipped with Leica DFC480 camera (Leica Microsystems, Germany). Image analysis was performed using ImageJ software.

### Statistical Analysis

Graphpad prism was used for statistical analyses. Data normality was assessed using Kolmogorov Smirnoff tests. In all instances where raw data were not normally distributed, log transformation was sufficient to normalise data. Normally distributed data were then analysed using one way ANOVA to determine if significant differences were present across treatments, followed by Bonferroni post-hoc test where ANOVA was statistically significant, and where analyses compared only planned treatments (Imatinib experiments). All mean±sem and p values are listed in Table S1.

## Results

### Cisplatin and Doxorubicin Induce Follicle Loss and Unhealthy Follicles

**Figure 1:**
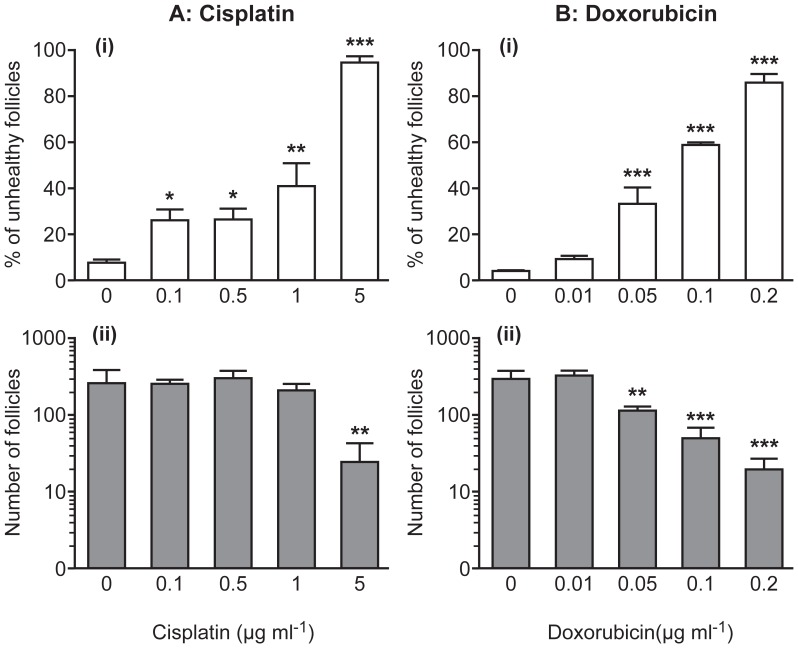
Cisplatin and doxorubicin both lead to loss of follicle health and a reduction in follicle numbers. (**A**) Cisplatin; (**B**) Doxorubicin: (**i**) Percentage of unhealthy follicles (clear); and (**ii**) total number of follicles (shaded) in each ovary. Bars denote mean+sem; n = 5 for all groups, stars denote significant differences relative to control (*p<0.05, **p<0.01, ***p<0.001).

Newborn ovaries were cultured for six days in control medium or exposed to cisplatin or doxorubicin only on Day 2 of culture (Fig. 1). Over the six days, most primordial follicles initiated growth to the transitional or primary stage, with few reaching the secondary stage. The proportion of follicles deemed morphological unhealthy significantly increased with dosage for both cisplatin and doxorubicin (Fig. 1Ai and Bi). To assess whether drug treatment led to follicle loss, total follicle number was calculated from histological analyses and log transformed for normality. Both cisplatin and doxorubicin caused significant follicle loss, but with different patterns of dose response (Fig. 1Aii and Bii). Cisplatin induced significant loss of follicles only at the highest concentration (5 µg ml^−1^; p<0.01, n = 5), which induced poor health in almost all follicles (Fig. 1A). In contrast doxorubicin resulted in a significant decrease in follicle number (p<0.05, n = 5) even at a dose which induced poor health in only around 30–35% of follicles (0.05 µg ml^−1^; Fig. 1B).

### Cisplatin and Doxorubicin Target Different Follicle Stages

**Figure 2:**
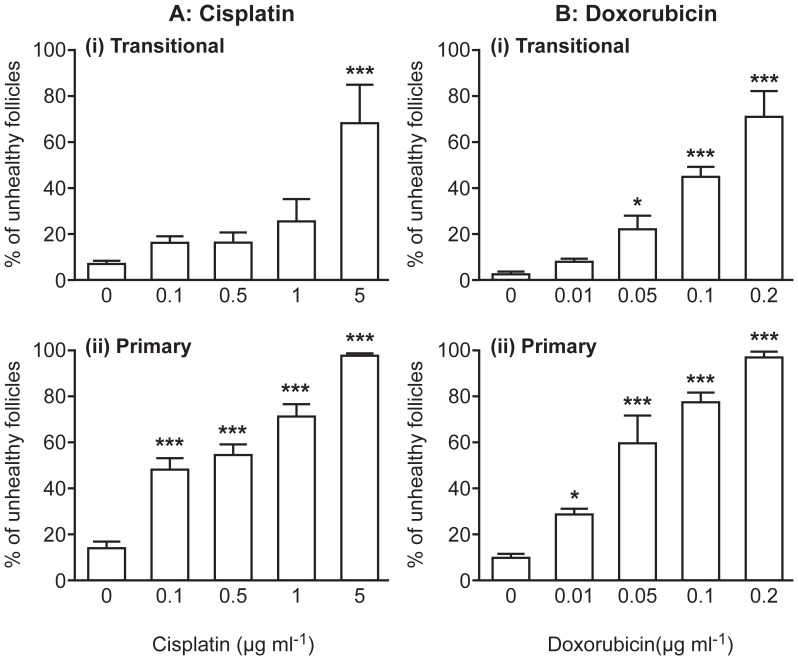
Cisplatin and doxorubicin affect different follicle classes. Follicles were classified as morphologically unhealthy in cisplatin and doxorubicin treated ovaries according to follicle type. Effect of (**A**) Cisplatin or (**B**) Doxorubicin on the percentage of transitional and primary follicles classified as morphologically unhealthy. Bars denote mean+sem; n = 5 for all groups, stars denote significant differences relative to control (*p<0.05, **p<0.01, ***p<0.001).

As both drug treatments led to increased numbers of unhealthy follicles, they were further categorized to see if a particular follicle class was affected by each drug (Fig. 2). Both drugs caused a reduction in primordial follicle number to the point where, at the highest doses there were insufficient present to meaningfully analyse primordial follicle health (mean±sem number of primordial follicles in 5 µg ml^−1^ cisplatin group = 4±4, and in 0.2 µg ml^−1^ doxorubicin group = 1±1, vs control = 60±20; n = 5). Primordial follicle health was, therefore, assessed in a separate study (see below). Further analysis was possible, though, for transitional and primary follicles. Cisplatin caused a significant increase in the percentage of morphologically unhealthy transitional follicles only at the highest dose of cisplatin used (5 µg ml^−1^; p<0.001; n = 5), while there were a significant percentage of unhealthy primary follicles at all doses (Fig. 2A). In contrast, doxorubicin caused a significant increase in the percentage of both transitional and primary follicles classified as morphologically unhealthy from the second lowest dose (p<0.05 for transitional, p<0.001 for primary; n = 5; Fig. 2B).

### Cisplatin and Doxorubicin affect Different Ovarian Cell Types

**Figure 3:**
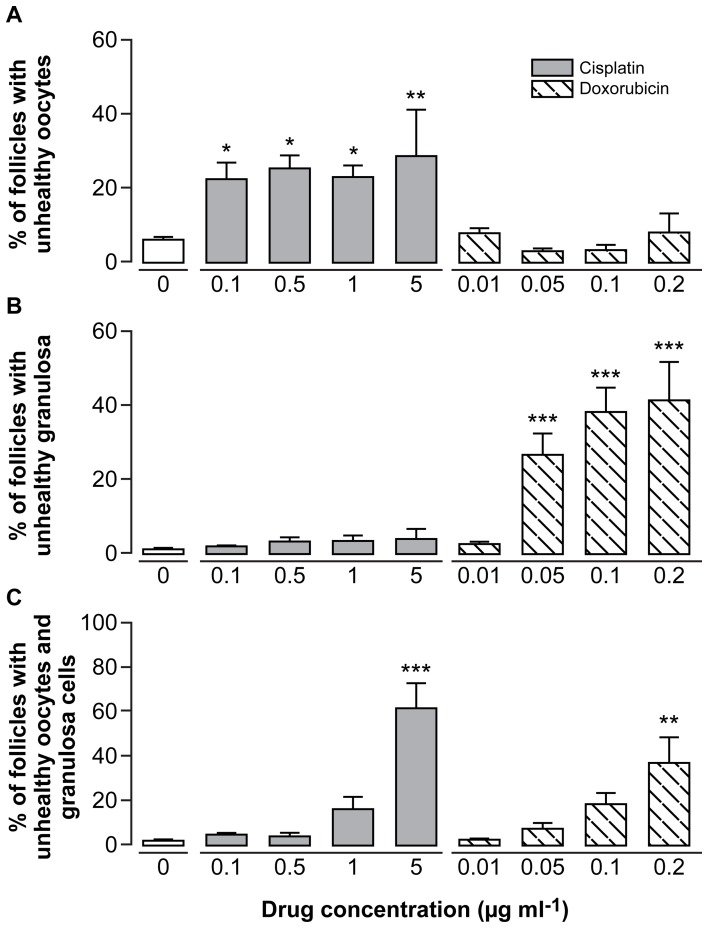
Cisplatin and doxorubicin affect different follicular cell types. Ovaries were treated with Cisplatin or Doxorubicin. All unhealthy transitional and primary follicles were further categorized as unhealthy due to: (**A**) poor oocyte health; (**B**) poor granulosa cell health; or (**C**) both. Bars denote mean+sem; n = 5 for all groups, stars denote significant differences relative to control (*p<0.05, **p<0.01, ***p<0.001).

In order to determine which specific follicular cell type was targeted by the two drugs, follicles were further classified as unhealthy due to: (a) the oocyte only; (b) granulosa cells only; or (c) both the oocyte and granulosa cells. In ovaries treated with cisplatin, unhealthy follicles were classified as such primarily due to oocyte health (Fig. 3A), with significant increases in the percentage of follicles with morphologically unhealthy oocytes seen at all doses of cisplatin used (p<0.05 for the lowest doses, p<0.01 for 5 µg ml^−1^; n = 5). In marked contrast, doxorubicin primarily induced follicles classified as unhealthy due to the granulosa cell health (Fig. 3B), with significant increases in the percentage of follicles with morphologically unhealthy granulosa cells seen at the three highest doses (p<0.001 for all three doses; n = 5). For both drugs, follicles in which both the oocyte and granulosa cells were unhealthy were rarely seen except at the two highest doses (Fig. 3C; p<0.001 for 5 µg ml^−1^ cisplatin, p<0.01 for 0.2 µg ml^−1^ doxorubicin; n = 5).

### Cisplatin and Doxorubicin do not Increase the Number of TUNEL-positive Primordial Follicles

**Figure 4:**
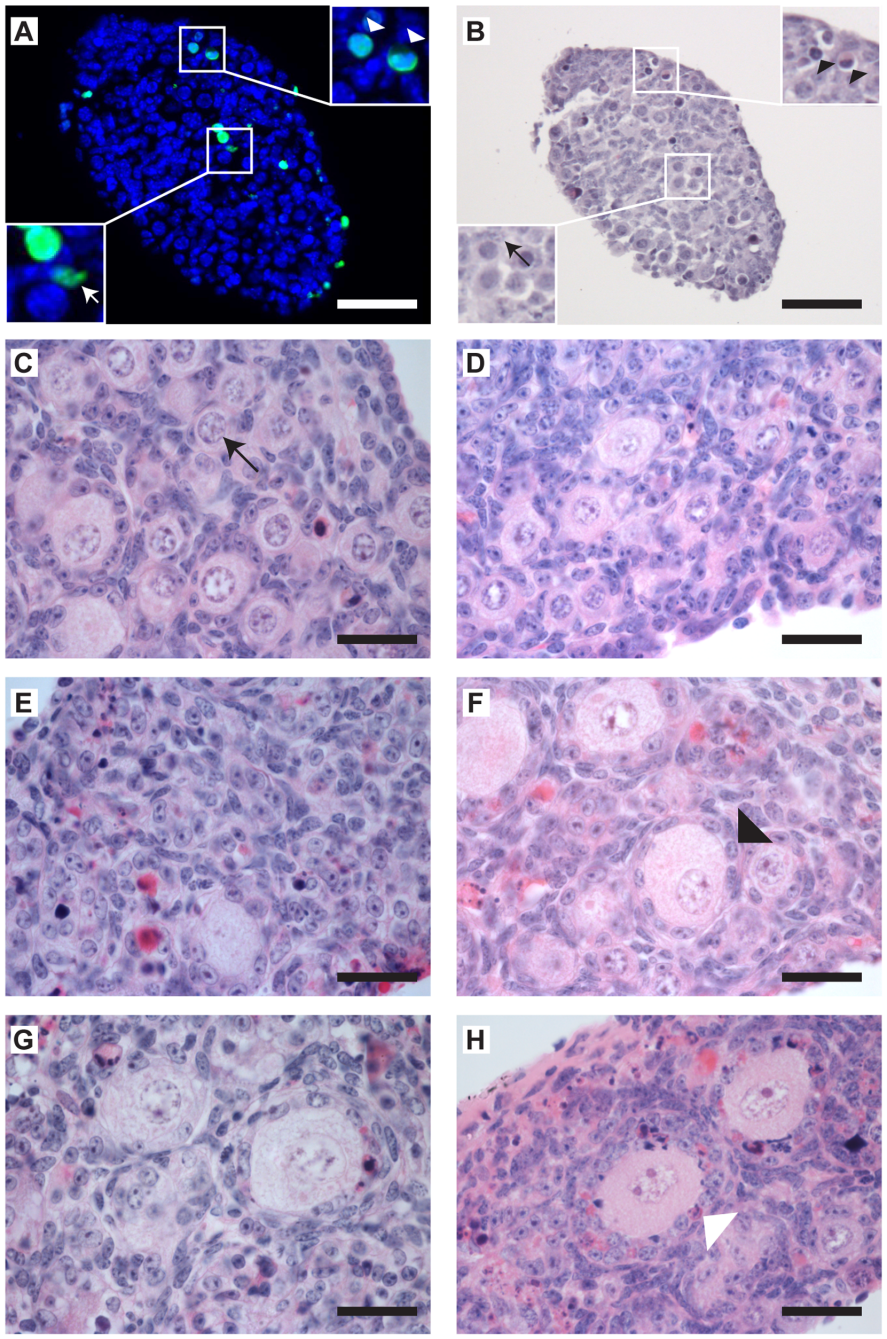
Representative histological sections of cultured mouse ovaries. (**A, B**)**:** TUNEL-analysis to determine apoptosis in primordial follicles. (**A**)**:** ovary section labelled with TUNEL reaction (green) and counterstained with DAPI (blue). Inset top- and bottom-right magnification images are of the respective framed areas, illustrating examples of TUNEL positive oocytes (white arrowheads) and TUNEL positive granulosa cell (white arrow) within follicles identified as at the primordial stage in (B). (**B**)**:** section in (A) subsequently stained with haematoxylin and eosin. Inset sections in (A) correspond here to degenerated oocytes (black arrowheads) and degenerated surrounding granulosa cells (black arrow) within primordial follicles. Scale bar = 50 µm. (**C–H**)**:** Photomicrographs of haemotoxylin and eosin stained sections from ovaries treated with (**C**) control, (**D**) 3 µg ml^−1^ imatinib, (**E**) 0.5 µg ml^−1^ cisplatin, (**F**) cisplatin and imatinib co-treatment, (**G**) 0.05 µg ml^−1^ doxorubicin and (**H**) doxorubicin and imatinib co-treatment. Scale bars represent 25 µm. Examples of a healthy primordial follicle (arrow), healthy growing follicle (black arrowhead) and unhealthy growing follicle (white arrowhead) are shown.

**Figure 5:**
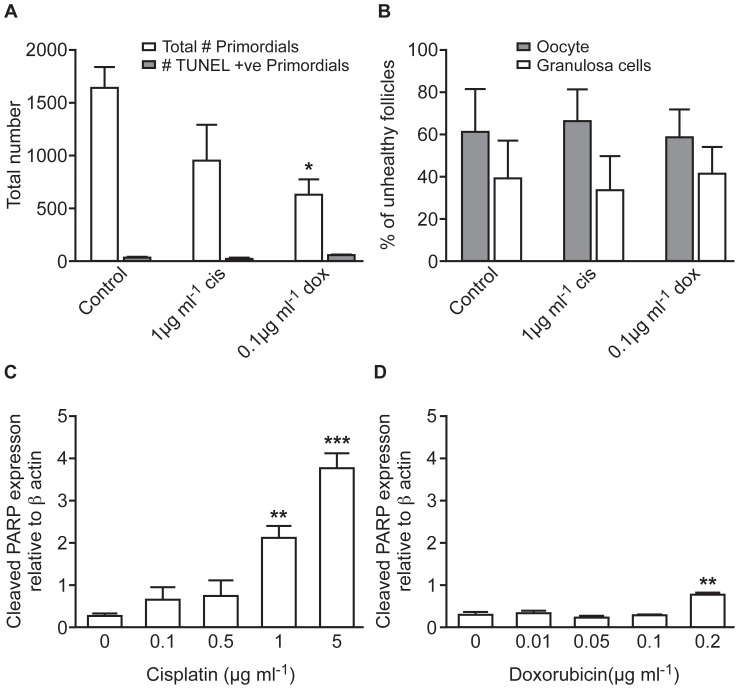
Effect of cisplatin and doxorubicin on primordial follicles, and on expression of cleaved PARP. (**A, B**)**:** Analysis of TUNEL-positive cells within primordial follicles. (**A**)**:** Total number of primordial follicles, and number of primordial follicles containing TUNEL-positive cells, in ovaries treated with cisplatin or doxorubicin. (**B**)**:** TUNEL-positive primordial follicles further categorised into percentage in which the oocyte or the granulosa cells stained positive. Bars denote mean+sem; n = 4–5; stars denote significant differences relative to control (**P<0.01). (**C, D**)**:** Cleaved PARP expression in cisplatin and doxorubicin treated ovaries. Protein expression of cleaved PARP relative to β actin (loading control) in whole newborn ovaries following 24 h of (**C**)**:** cisplatin or (**D**)**:** doxorubicin treatment. Examples of Western blots are shown in Supporting Information, Figure S3. Bars denote mean+sem; n = 3; stars denote significant differences relative to control (*p<0.05, **p<0.01, ***p<0.001).

After six days of culture, ovaries previously treated with high doses of chemotherapy drugs contained few primordial follicles (see above). Since a higher percentage of follicles are at the primordial stage earlier in the culture process (see Figure S1), apoptosis was analysed in ovaries immediately following drug exposure at the end of Day 2. TUNEL reaction was then carried out to identify apoptotic cells, with primordial follicles subsequently identified after co-staining with haematoxylin and eosin (Fig. 4A,B). Primordial follicle number present after treatment with 0.1 µg ml^−1^ doxorubicin was significantly lower than in control ovaries (p<0.05, n = 4–5), with a non-significant (p>0.05) decrease in the number present following treatment with 1 µg ml^−1^ cisplatin (n = 4–5; Fig. 5A). However, drug-exposure did not affect the number of primordial follicles positive for TUNEL staining (mean±sem in control group = 35±8, in doxorubicin group = 43±24, and in cisplatin group = 33±11; n = 4–5; Fig. 5A), which likewise was unaffected by the culture process (see Figure S2). Similarly, there was no change to the oocyte or granulosa cell distribution of such TUNEL-positive cells within the primordial follicles following either drug treatment (Fig. 5B). Together, results provide no evidence for primordial follicle loss in response to chemotherapy drug-exposure being due to apoptosis.

### Cell Death Pathway

Expression of the apoptosis marker cleaved PARP was analyzed using Western blotting to examine how the two drugs induced apoptosis (see Figure S3 for example of Western blots). PARP is a DNA repair protein cleaved in mid/late stage apoptosis. Cisplatin resulted in a significant dose-dependent increase in cleaved PARP expression, with the highest dose increasing expression 16-fold (p<0.001, Fig. 5C,D; n = 3). In contrast, doxorubicin had little effect, only significantly increasing cleaved PARP expression at the highest dose, and even then inducing only a 3-fold increase in expression (p<0.01, n = 3). This difference in cleaved PARP expression following exposure to the two drugs is despite the finding that the highest doses of both drugs induced loss of almost all follicles in both cases (Figs. 1Aii, Bii, 5C,D).

### Imatinib Cotreatment Protects Follicles against Cisplatin but not Doxorubicin

**Figure 6:**
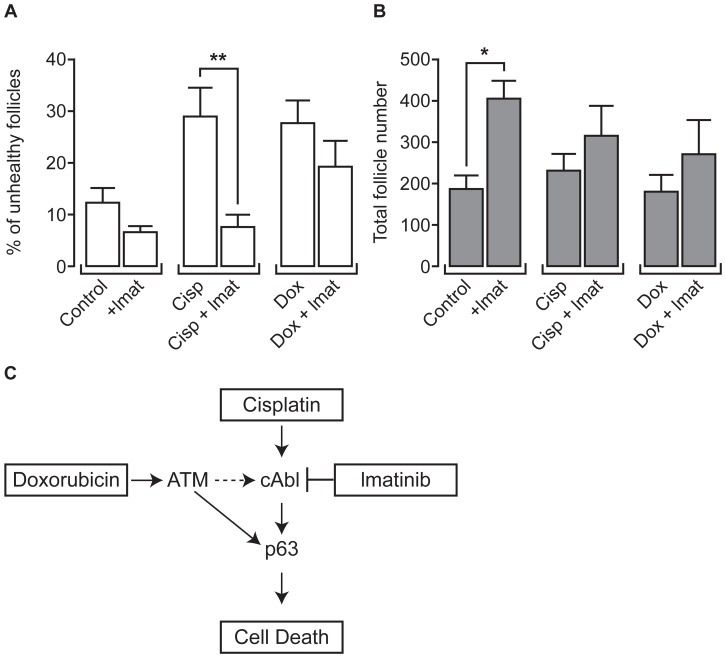
Imatinib co-treatment with cisplatin, but not doxorubicin, rescues follicle health. Control, cisplatin-treated (0.5 µg ml^−1^) and doxorubicin-treated (0.05 µg ml^−1^) cultured ovaries were cultured in the presence or absence of imatinib. (**A**)**:** Percentage of unhealthy follicles (clear); (**B**)**:** Total number of follicles (shaded). Bars denote mean+sem; n = 7 for all groups, stars denote significant differences relative to control (**p<0.01). (**C**)**:** Pathway by which imatinib could protect the ovary against the damaging effect of cisplatin more effectively than it could against the damaging effect of doxorubicin.

Since recent evidence has suggested that the tyrosine kinase inhibitor imatinib can alleviate cisplatin-induced ovarian damage (17), ovaries were exposed to imatinib using the present model, to compare its ability to protect the ovary against damage by both cisplatin and doxorubicin. Ovaries were treated with either cisplatin (0.5 µg ml^−1^) or doxorubicin (0.05 µg ml^−1^), with those mid-range doses chosen as both induced the appearance of a similar percentage of unhealthy follicles (around 30%), without causing the extensive damage to the ovary found after exposure to the highest doses. Drug exposure was limited to Day 2 of culture as in previous experiments, with imatinib (3 µg ml^−1^) present throughout Days 1–3 of culture to maximize any potential protective capacity of the drug. Imatinib treatment alone led to a small reduction in the percentage of unhealthy follicles when compared to control although this was not significant (n = 7; Figs. 4C,D, 6A). Imatinib had a clear protective effect against damage from cisplatin, reducing the percentage of unhealthy follicles by 21% (p<0.01; n = 7; Figs. 4E,F, 6A). Imatinib tended to lead to a reduction in the percentage of unhealthy follicles present during exposure to doxorubicin (9% reduction), but this was not significant (Bonferroni adjusted p = 0.6: n = 7; Figs. 4G,H, 6A). The presence of imatinib alone also lead to a higher number of follicles present in the ovary at the end of culture (p<0.05; n = 7): the same trend occurred in the cisplatin- and doxorubicin- treated cultures but was not significant in either case (p>0.05 for both: Fig. 6B).

## Discussion

Chemotherapy treatment has long been associated with POF and infertility in premenopausal patients. It is often assumed that chemotherapy drugs directly damage oocytes in the primordial follicle reserve and that it is this loss that leads to POF. There is, however, little available evidence for this: instead, chemotherapeutic drugs could primarily damage the growing population of ovarian follicles leading to increased growth activation of primordial follicles and thus premature depletion [15], while initial site of damage could be either oocytes and/or somatic cells [8]. The culture system used here allows analysis of the early stages of follicle development in a highly physiological and controlled environment. Our results show that cisplatin and doxorubicin lead to a different pattern of follicle loss/damage and that they act through different cellular mechanisms. Furthermore, imatinib provided protection to follicles from cisplatin but not doxorubicin.

Cisplatin and doxorubicin are commonly used in cancer treatment of premenopausal women and both have been linked to follicle loss and POF. Data here are consistent with these findings, showing an increase in unhealthy follicles, and a reduction in total follicle number, following treatment with either cisplatin or doxorubicin. Both drugs were used in doses within the therapeutic range for patients, which are around 0.5–1 µg ml^−1^ for cisplatin [31], [32] and about 0.02–0.6 µg ml^−1^ for doxorubicin [33], [34]. As the culture system here supports primordial follicle formation, activation and maturation through to the primary stage, the effect of chemotherapy drugs on very specific populations of follicles could be assessed. Administration of chemotherapeutic agents in culture also allowed both dosage and duration of exposure to be tightly controlled.

Loss of the primordial follicle pool via chemotherapy-induced damage, whether through direct or indirect action, is an important consideration, as this loss will directly lead to POF. Here, few primordial follicles were left after exposure to high levels of cisplatin or doxorubicin, but TUNEL analysis failed to find evidence of increased apoptosis within the primordial follicles. The simplest explanation for this is that the reduction in primordial follicle numbers after drug exposure is due to growth initiation, presumably as a result of the death of growing follicles and thus loss of local growth initiation inhibiting factors, such as anti-Müllerian hormone (AMH), [35]. These results support those of [12], who treated mice *in vivo* with doxorubicin and analysed ovaries following 24 hours of exposure; they saw little or no TUNEL-positive cells in primordial follicles, while TUNEL-positive cells were seen in secondary, preantral and antral follicles.

In contrast to the indirect effect of the drugs on primordial follicles, direct damage to later stages of follicles was clear, with cisplatin treatment mainly damaging primary follicles, while doxorubicin treatment led to unhealthy follicles at both transitional and primary stages, indicating a greater vulnerability of follicles to doxorubicin as soon as they leave the primordial stage. Depletion in both primary and primordial follicles has previously been demonstrated in mouse pups injected with cisplatin [17].

Cisplatin causes cell death primarily through DNA crosslinking and adduct formation [36]. In cisplatin-treated ovaries, the oocyte was the primary reason for a follicle being classified as morphologically unhealthy, suggesting that cisplatin directly targets the germ cell. Given the importance from an evolutionary prospective of protecting the integrity of the germ line, it may be that oocytes are particularly vulnerable to agents causing overwhelming DNA damage. It is well established that the oocyte is particularly susceptible to cell death following DNA damage caused by radiotherapy [37]. It is perhaps not that surprising, therefore, that oocytes would also be highly susceptible to the damaging effects of cisplatin. The preferential site of cisplatin toxicity being the oocyte is perhaps also why follicle health was not reduced in follicles on initiation of growth (ie transitional follicles), but manifested at the slightly later primary stage.

Doxorubicin can cause DNA damage and inhibit topoisomerase enzymes, both of which inhibit DNA replication and cell division. In contrast to the effect of cisplatin, results here show that doxorubicin primarily targeted the granulosa cells of follicles. An *in vivo* study which used 4 week old female mice injected with a single dose of doxorubicin also found an increase in granulosa cells which stained positive for caspase 3, indicating apoptosis [12]. In contrast to oocytes, granulosa cells are mitotically active, which may explain their vulnerability to these types of chemotherapy agents. Doxorubicin induces apoptosis in mature (MII) oocytes *in vitro* [9], [10] and a recent study has shown that oocytes collected from antral follicles and cultured *in vitro* are also highly susceptible to such damage [38]. The oocytes examined here were not mature, but instead contained within non-growing or early-growing follicles, which may explain why they are less sensitive to doxorubicin in this system. This model is, though, possibly more relevant to patients, since the oocytes required to maintain long term fertility will also be contained within non-growing follicles. A recent study of human primordial follicles treated *in vitro* with doxorubicin showed damage to both oocytes and granulosa cells, although that study used much higher concentrations of doxorubicin than were used here, with levels in this study encompassing those found in the serum of patients [19], [33], [34].

PARP is a DNA repair protein which detects the presence of single and double strand DNA breaks. When DNA damage is minor, PARP activates enzymatic machinery such as DNA polymerase and ligase, allowing repair of these breaks [39]. Where DNA damage is extensive, activation of PARP can lead to either necrotic or apoptotic cell death. PARP can be cleaved by caspase-dependent or independent mechanisms, leading to cell death. Although there is some evidence that PARP cleavage is not essential to the process of apoptosis, it is generally considered one of the hallmarks of apoptotic cell death [40]. Cisplatin caused a dose-dependent and robust increase in cleaved PARP expression, in marked contrast to doxorubicin which led to only a small increase in expression and only at the highest dose used, further evidence that cisplatin and doxorubicin are causing cell death through quite different mechanisms.

The fact that the two cytotoxic agents cause POF in different ways should not be surprising. The mechanism of action and toxicities of different chemotherapeutic agents are discrete and while it may be that some of the doxorubicin effect on the growing follicle pool is due to a cytotoxic effect on the dividing granulosa cells, our data suggest this is not the cause of cisplatin-induced damage. This raises the question of how a cytotoxic agent such as cisplatin affects non-dividing cells. It is, however, known that neuropathy is one of the main toxicities of cisplatin therapy despite the fact that neurones are not actively dividing. The mechanism by which cisplatin cause neurotoxocity is not precisely understood, but at the cellular level cisplatin affects the metabolic function of the neuron [41] with the effect partly mediated through the formation of platinum-DNA-protein crosslinks [42]. It has also been demonstrated *in vitro* and *in vivo* that the formation of platinum-DNA adducts in the dorsal root ganglion can result in neuronal apoptosis, despite the fact that these neurones are not actively dividing [43]. It is very possible that these mechanisms of affecting non-dividing cells could also be responsible for the oocyte toxicity that was seen in our study for cisplatin but not doxorubicin. Neuroprotection during cisplatin treatment has also been demonstrated following antioxidant treatment [44], [45], indicating that cisplatin may be causing neuronal cell death through oxidative stress. The role of oxidative stress in the ovary with regards to primordial and primary follicles is currently unclear but is another potential mechanism though which cisplatin could cause follicle loss [46].

Any damage to ovarian stromal cell health could also negatively impact on follicle reserve. Whilst some studies have suggested that chemotherapeutic drugs could have a negative effect on the health of the stroma ([47], [48], no consistent quantifiable increase in stromal pyknosis was found here, with some scattered pyknotic cells evident, but their presence was very inconsistent among treatments (results not shown).

Recent work has suggested that the tyrosine kinase inhibitor imatinib can reduce the toxic effect of cisplatin on the ovary through its inhibition of c-Abl [17]. Results here provide clear evidence of a reduction by imatinib of the adverse effect of cisplatin on follicle health, and show that protection is specific, with no significant protection found against doxorubicin-induced damage. Imatinib is thought to provide ovarian protection against cisplatin damage by inhibiting c-Abl, a tyrosine kinase which promotes accumulation of p63, the oocyte-specific homologue of p53: p63 in turn activates cell death following high levels of DNA damage [17], [49], [50]. In contrast, doxorubicin upregulates ataxia telangiectasia mutated (ATM) in the ovary, which is another activator of cell death in the presence of high levels of DNA damage [19]. In that study, the authors suggest that ATM can cause upregulation of p63 not only through independent pathways, but also via a c-Abl-dependant pathway, perhaps explaining the trend towards a protective effect of imatinib found here (Fig. 6C).

Imatinib treatment alone did not have a deleterious effect on the ovary, in agreement with some recent work [24], [51] although in contrast to Kerr *et al* [25] who found no protective effect of imatinib against cisplatin-induced damage, instead showing imatinib-induced ovarian damage [25]. The difference between these studies is possibly due to differences in drug dosages: 10 µM imatinib, 20 µM cisplatin in Kerr *et al* [25]; 1 µM imatinib and 7.5 µM cisplatin in Maiani *et al* [24] and approximately 5 µM imatinib and 1.67 µM cisplatin here. Interestingly, imatinib alone increased total follicle number here, due either to inhibition of follicle death and/or to stimulation of follicle formation: ovaries were treated with imatinib at the start of the culture period, when follicle formation still continues *in vivo* through to postnatal day 3, with follicle number also increasing in the equivalent period *in vitro* (Figure S2). Tyrosine kinase signalling has previously been implicated to play a positive role in both, leading to increased primordial follicle formation [52] as well as promoting the survival of primordial germ cells [53].

In summary, data here show that cisplatin and doxorubicin both induced follicle loss in cultured mouse ovaries. There was no evidence of direct effects of either drug on primordial follicle health, with follicular atresia increased only from within the growing follicle pool, but each drug induced specific patterns of damage across and within follicles. One consequence of the different actions of these two drugs is that any treatments designed to protect the ovary from chemotherapy-induced damage may have to be tailored to the specific drug regimens used; this concept is confirmed by the selective protection afforded by imatinib mesylate against damage from cisplatin but not from doxorubicin.

## Supporting Information

Figure S1 Follicle numbers and composition in control ovaries cultured for up to six days.
(PDF)

Figure S2 Comparison of TUNEL-positive primordial follicles in uncultured and cultured ovaries.
(PDF)

Figure S3 Examples of Western blots for the detection of Cleaved PARP.
(PDF)

Table S1 Individual means, SEMs and p values for data in Figures.
(PDF)
