# Supplementary material for: Cisplatin and Doxorubicin Induce Distinct Mechanisms of Ovarian Follicle Loss; Imatinib Provides Selective Protection Only against Cisplatin
Source: PLoS One. 2013 Jul 29;8(7):e70117. doi: 10.1371/journal.pone.0070117 (PMC3726485; doi:10.1371/journal.pone.0070117)

Cisplatin and Doxorubicin induce distinct mechanisms of ovarian follicle loss; imatinib provides selective protection only against cisplatin.

Morgan, Lopes, Gourley, Anderson and Spears.

Supporting Information.

Figure S1

Follicle numbers and composition in control ovaries cultured for up to six days.

Newborn female mice were culled and ovaries dissected out into Leibovitz L-15 dissection medium supplemented with 3mg ml^{-1} bovine serum albumin. Ovaries were either fixed immediately (n= 4) or cultured on Whatman Nucleopore membranes floating on 1ml α-MEM medium supplemented with 3mg ml^{-1} bovine serum albumin, in a 24 well plate incubated at 37°C, 5% CO_2. Ovaries were removed from culture daily (day 1, n=3; day 2, n=3; day 3, n=4; day 4, n=4; day 5, n=3; day 6, n=5). Ovaries were then sectioned, stained with haematoxylin and eosin and every sixth section was analysed as in the paper.

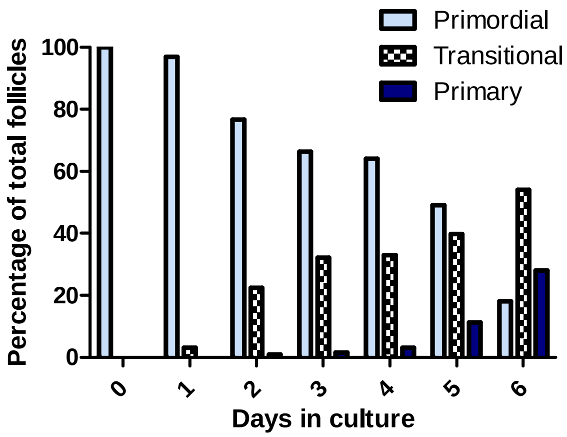

Supplement: Figure S1 — Follicle numbers and composition in control ovaries cultured for up to six days. (PDF) [file pone.0070117.s001.pdf]
