# Supplementary material for: Cisplatin and Doxorubicin Induce Distinct Mechanisms of Ovarian Follicle Loss; Imatinib Provides Selective Protection Only against Cisplatin
Source: PLoS One. 2013 Jul 29;8(7):e70117. doi: 10.1371/journal.pone.0070117 (PMC3726485; doi:10.1371/journal.pone.0070117)

Cisplatin and Doxorubicin induce distinct mechanisms of ovarian follicle loss; imatinib provides selective protection only against cisplatin.

Morgan, Lopes, Gourley, Anderson and Spears.

<u>Supporting Information.</u>

<u>Figure S2</u>

Comparison of TUNEL-positive primordial follicles in uncultured and cultured ovaries.

Ovaries were collected from P0 and P2 mice (n=3 for each), and processed for TUNEL analysis, as in the paper. Results were compared with ovaries from P0 mice cultured for two days, from Control, Cisplatin-treated and Doxorubicin-treated ovaries (data from Figure 4 of the paper). Data were analysed using one way ANOVA to determine if significant differences were present across treatments, followed by Student's unpaired t tests where ANOVA was statistically significant (this was the case only for total number of primordial follicles).

(**A**): Total number of primordial follicles and number of TUNEL-positive primordial follicles.
(**B**):Percentage of primordial follicles which were TUNEL-positive.
Bars denote mean+sem; stars denote significant differences relative to control (*$p<0.05$, ***$p<0.0001$).

Total number of primordial follicles increased significantly after two days development *in vivo* (P0 compared with P2) and *in vitro* (P0 compared with cultured Control group). No significant difference was found between uncultured and cultured ovaries in the (a) number or (b) percentage of TUNEL-positive primordial follicles.

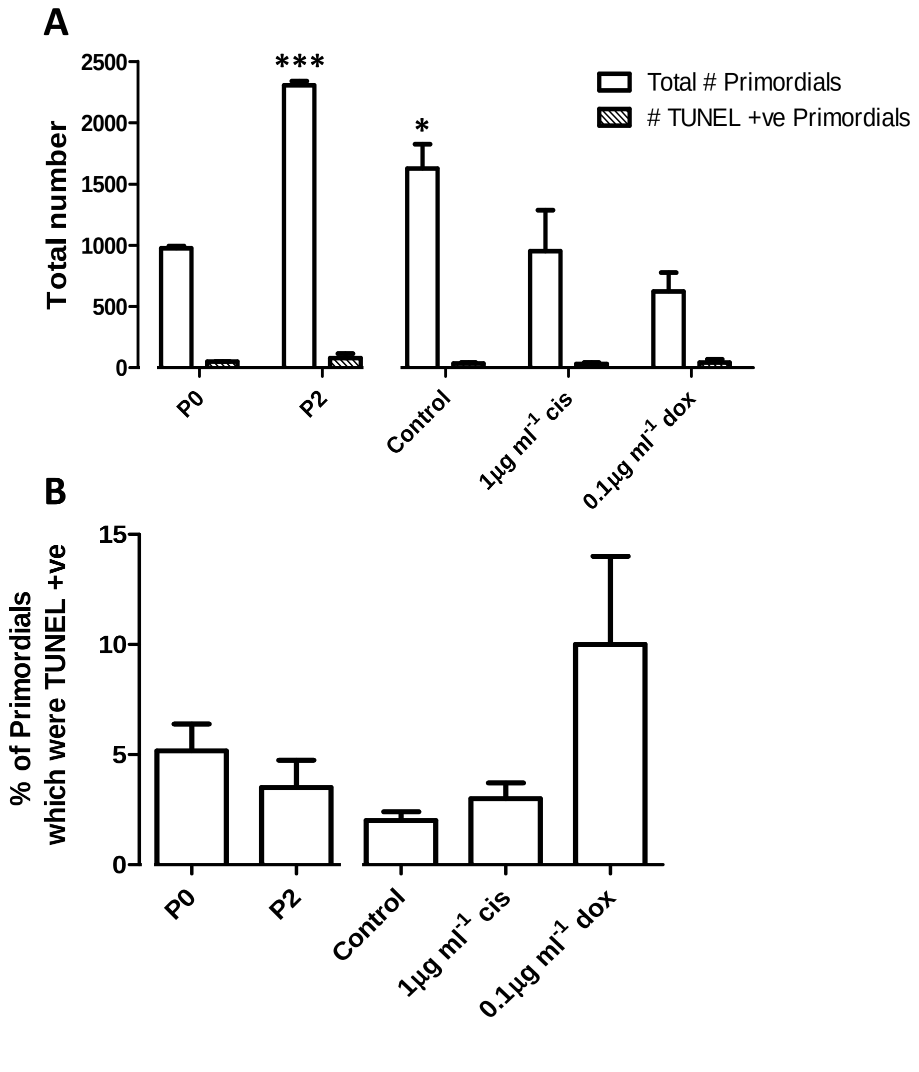

Supplement: Figure S2 — Comparison of TUNEL-positive primordial follicles in uncultured and cultured ovaries. (PDF) [file pone.0070117.s002.pdf]
