# Supplementary material for: Cisplatin and Doxorubicin Induce Distinct Mechanisms of Ovarian Follicle Loss; Imatinib Provides Selective Protection Only against Cisplatin
Source: PLoS One. 2013 Jul 29;8(7):e70117. doi: 10.1371/journal.pone.0070117 (PMC3726485; doi:10.1371/journal.pone.0070117)

Cisplatin and Doxorubicin induce distinct mechanisms of ovarian follicle loss; imatinib provides selective protection only against cisplatin.

Morgan, Lopes, Gourley, Anderson and Spears.

Supporting Information.

Figure S3

Examples of Western blots for the detection of Cleaved PARP.

Approximately 10µg of protein from each ovary was loaded onto a 7% acrylamide gel and run at 30mA at room temperature. Protein was transferred onto a nitrocellulose membrane and blocked using 5% powdered milk (w/v) in phosphate buffered saline (PBS, pH7.3, 160mM NaCl, 3mM KCl, 8mM Na_2HPO_4, 1mM KH_2PO_4). PARP rabbit polyclonal antibody (New England Biolabs, Hertfordshire UK) was added at 1:1000 dilution: this antibody to PARP detects both the full length (116kD) and cleaved (89kD) form. β-actin rabbit polyclonal antibody (Abcam, Cambridge UK) was added at 1:5000 dilution as a loading control (β-actin giving a 45kD band). Membranes were then incubated overnight at 4°C. After washing in PBS, membranes were incubated with Alexafluor anti-rabbit 750 (Invitrogen, UK) at 1:2000 for 1h, membranes re-washed and dried, and imaging and analysis carried out using a Li-cor scanner and Odyssey v1.2 software (Li-cor Biosciences, US). 89kD bands were analysed to determine expression of Cleaved PARP, with 45kD bands analysed to determine expression of β-actin, for results shown in Figure 5C,D of paper.

Western blots below are of ovaries treated with varying concentrations of (**A**) cisplatin; and (**B**) doxorubicin.

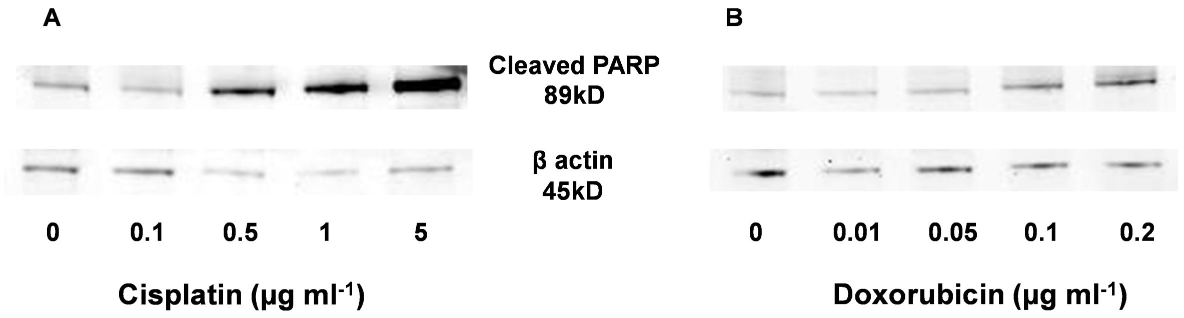

Supplement: Figure S3 — Examples of Western blots for the detection of Cleaved PARP. (PDF) [file pone.0070117.s003.pdf]
