# Supplementary material for: Cisplatin and Doxorubicin Induce Distinct Mechanisms of Ovarian Follicle Loss; Imatinib Provides Selective Protection Only against Cisplatin
Source: PLoS One. 2013 Jul 29;8(7):e70117. doi: 10.1371/journal.pone.0070117 (PMC3726485; doi:10.1371/journal.pone.0070117)
Supplement: Table S1 — Individual means, SEMs and p values for data in Figures. (PDF) [file pone.0070117.s004.pdf]

Cisplatin and Doxorubicin induce distinct mechanisms of ovarian follicle loss; imatinib provides selective protection only against cisplatin.

Morgan, Lopes, Gourley, Anderson and Spears.

Supporting Information.

Table S1.

Individual means, SEMs and p values for data in Figures

	**Mean±SEM**	**p value**	**Mean±SEM**	**p value**
	Figure 1A(i)		*Figure 1A(ii)*	
Control	7±2	-	335±95	-
0.1µg ml^{-1} cisplatin	26±5	<0.05	265±29	ns
0.5µg ml^{-1} cisplatin	26±5	<0.05	330±70	ns
1µg ml^{-1} cisplatin	41±10	<0.01	230±48	ns
5µg ml^{-1} cisplatin	94±3	<0.001	43±21	<0.01
	Figure 1B(i)		*Figure 1B(ii)*	
Control	3±1	-	315±76	-
0.01µg ml^{-1} doxorubicin	9±2	ns	333±52	ns
0.05µg ml^{-1} doxorubicin	33±7	<0.001	111±12	<0.05
0.1µg ml^{-1} doxorubicin	58±1	<0.001	57±16	<0.01
0.2µg ml^{-1} doxorubicin	85±4	<0.001	24±7	<0.001
	Figure 2A(i)		*Figure 2A(ii)*	
Control	7±2	-	13±4	-
0.1µg ml^{-1} cisplatin	16±4	ns	49±5	<0.001
0.5µg ml^{-1} cisplatin	16±5	ns	55±5	<0.001
1µg ml^{-1} cisplatin	25±10	ns	72±5	<0.001
5µg ml^{-1} cisplatin	68±18	<0.001	99±1	<0.001
	Fig 2B(i)		*Fig 2B(ii)*	
Control	2±1	-	9±2	
0.01µg ml^{-1} doxorubicin	7±1	ns	28±3	<0.05
0.05µg ml^{-1} doxorubicin	22±6	<0.05	60±12	<0.001
0.1µg ml^{-1} doxorubicin	44±5	<0.001	78±4	<0.001
0.2µg ml^{-1} doxorubicin	70±12	<0.001	98±2	<0.001
	Fig 3A		*Fig 3B*	
Control	6±1	-	1±1	-
0.1µg ml^{-1} cisplatin	22±5	<0.05	2±1	ns
0.5µg ml^{-1} cisplatin	25±4	<0.05	3±1	ns
1µg ml^{-1} cisplatin	23±3	<0.05	3±1	ns
5µg ml^{-1} cisplatin	28±13	<0.01	4±3	ns
0.01µg ml^{-1} doxorubicin	8±2	ns	2±1	ns
0.05µg ml^{-1} doxorubicin	3±1	ns	27±6	<0.001
0.1µg ml^{-1} doxorubicin	3±1	ns	38±7	<0.001
0.2µg ml^{-1} doxorubicin	8±12	ns	41±10	<0.001

	Fig 3C			
Control	2±1	-		
0.1µg ml^{-1} cisplatin	4±1	ns		
0.5µg ml^{-1} cisplatin	4±1	ns		
1µg ml^{-1} cisplatin	16±6	ns		
5µg ml^{-1} cisplatin	62±11	<0.001		
0.01µg ml^{-1} doxorubicin	2±1	ns		
0.05µg ml^{-1} doxorubicin	7±3	ns		
0.1µg ml^{-1} doxorubicin	18±5	ns		
0.2µg ml^{-1} doxorubicin	37±12	<0.01		
	Fig 5A			
Control	1626±198	-		
1µg ml^{-1} cisplatin	951±335	ns		
0. 1µg ml^{-1} doxorubicin	621±156	<0.05		
	Fig 5B			
	Oocyte	*Granulosa*		
Control	61±20	39±13		
1µg ml^{-1} cisplatin	65±16	35±15		
0. 1µg ml^{-1} doxorubicin	58±16	42±12		
	Fig 5C, D			
Control	0.23±0.06	-		
0.1µg ml^{-1} cisplatin	0.62±0.33	ns		
0.5µg ml^{-1} cisplatin	0.69±0.38	ns		
1µg ml^{-1} cisplatin	2.06±0.28	<0.01		
5µg ml^{-1} cisplatin	3.7±0.35	<0.001		
0.01µg ml^{-1} doxorubicin	0.31±0.10	ns		
0.05µg ml^{-1} doxorubicin	0.18±0.05	ns		
0.1µg ml^{-1} doxorubicin	0.23±0.10	ns		
0.2µg ml^{-1} doxorubicin	0.75±0.07	P<0.01		
	Fig 6A		*Fig 6B*	
Control	12±3	-	187±33	-
Imatinib	7±1	ns[#]	402±43	<0.05[#]
Cisplatin	29±5	-	228±43	-
Cisplatin+Imatinib	8±2	<0.01[#]	315±72	ns[#]
Doxorubicin	28±4	-	176±45	-
Doxorubicin+Imatinib	19±5	ns[#]	270±83	ns[#]

[#] p values from comparing each imatinib-exposure group to its control.
